# Methods to identify the unexplored diversity of microbial exopolysaccharides

**DOI:** 10.3389/fmicb.2015.00565

**Published:** 2015-06-09

**Authors:** Broder Rühmann, Jochen Schmid, Volker Sieber

**Keywords:** polysaccharide, screening, high throughput, carbohydrate fingerprint, colorimetric assays

## Abstract

Microbial exopolysaccharides (EPS) are a structurally very diverse class of molecules. A number of them have found their application in rather diverging fields that extend from medicine, food, and cosmetics on the one side to construction, drilling, and chemical industry on the other side. The analysis of microbial strains for their competence in polysaccharide production has therefore been a major issue in the past, especially in the search for new polysaccharide variants among natural strain isolates. Concerning the fact that nearly all microbes carry the genetic equipment for the production of polysaccharides under specific conditions, the naturally provided EPS portfolio seems to be still massively underexplored. Therefore, there is a need for high throughput screening techniques capable of identifying novel variants of bacterial EPS with properties superior to the already described ones, or even totally new ones. A great variety of different techniques has been used in screening approaches for identifying microorganisms that are producing EPS in substantial amounts. Mucoid growth is often the method of choice for visual identification of EPS producing strains. Depending on the thickening characteristics of the polysaccharide, observation of viscosity in culture broth can also be an option to evaluate EPS production. Precipitation with different alcohols represents a common detection, isolation, and purification method for many EPS. A more quantitative approach is found in the total carbohydrate content analysis, normally determined, e.g., by phenol-sulfuric-acid-method. In addition, recently a new and reliable method for the detailed analysis of the monomeric composition and the presence of rare sugars and sugar substitutions has become available, which could give a first hint of the polymer structure of unknown EPS. This minireview will compare available methods and novel techniques and discuss their benefits and disadvantages.

## Introduction

The global production of bacterial polymers is increasing rapidly, caused by the growing demand for biobased polymers. The natural variety of different exopolysaccharides (EPS) with specific properties has a huge potential for industrial utilization. Based on the Bacterial Carbohydrate Structure Data Base (Toukach et al., 2007) ca. Four hundred different EPS variants with different chemical structures have been published, of which some can be linked to specific strains or genera. Additionally many reports can be found, which describe microbes to be capable of producing EPS, without giving structural information. This impressively demonstrates the high diversity of naturally available EPS and the capacity for new variants to be of technical and commercial interest. Especially the growing demand of sustainable products further increases the need for the replacement of petro-based polymers such as polyacrylates or polyvinyl alcohol. An example of one of those new products is the biobased lubricant Berufluid^®^. Furthermore, in fields such as medicine (Colegrove, 1983; Costerton et al., 1999), cosmetics (Thibodeau, 2005; Prajapati et al., 2013), water treatment (Srinivasan, 2013), agriculture (Colegrove, 1983), enhanced oil recovery (Rau and Brandt, 1994), and construction chemistry (Schmidt et al., 2013) new and innovative EPS variants are used. In order to trap the full potential of EPS fast and reliable screening methods are important to identify novel EPS with innovative properties to enhance the field of applications. Here we describe the most common and publicly available screening approaches that have been used including the different methods for identification of EPS on which they are based and discuss their advantages and disadvantages (**Table 1**) as well as their compatibility for high-throughput (HT).

**Table 1:** Overview and description of different screening approaches including benefits and disadvantages.

Method/Reference	*Example of Screening Approach*/Description	Pros and Cons
**Detection of exopolysaccharides (EPS) producing phenotypes**
Colony morphology Ortega-Morales et al. (2007)	*Screening of 34 strains for biofilm formation:* – replication of isolates selection of mucoid (slimy) colonies (indication for their ability to produce exopolymeric substances) biomass scraped from the agar surface dissolving and centrifugation precipitation with two volumes cold ethanol dissolving and detection via phenol-sulfuric acid method	+ simple experimental setup and low cost + only small amounts needed preselection via visual observation (mucoid colonies) may result in many false negative only EPS which precipitate detected only determination of total carbohydrate content
Colony morphology Tallgren et al. (1999)	*Screening of 600 strains for EPS production from sugar beets* – first screening round: 170 out of 600 strains detected via slimy colony morphology 17 randomly chosen isolates centrifugation of liquid culture precipitation with two volumes isopropanol collection of precipitate by centrifugation and freeze dried hydrolysis: 1N H_2_SO_4_ for 60 min at 120°C – neutralization with 5 M sodium hydroxide – monosaccharide composition: via high performance anion exchange chromatography with pulsed amperometric detection (HPAEC-PAD)	+ preselection: simple experimental setup and low cost + detailed monosaccharide analysis of selected strains preselection via visual observation (mucoid colonies) may result in many false negatives randomly chosen isolates for further monosaccharide analysis only 10% of positive strains were analyzed not manageable in high throughput
**Agar-plate with dyes**
Aniline Blue Ma and Yin (2011)	*Screening for EPS producers in different environments* – interacts with β-(1-3)-glucans visual observation of colony color and morphology	+ manageable in high throughput and low cost fluorochrome Sinofluor is only an impurity in Aniline Blue
Calcofluor White Leigh et al. (1985)	*Screening of defective mutants in succinoglycan production:* – visual observation under UV-light Calcofluor-dark mutants are defective in EPS production.	+ manageable in high throughput and low cost + binding of Calcofluor White to succinoglycan and (1-4)- and (1-3)--glucans
Congo Red Darwish and Asfour (2013)	*Biofilm formation ability in Staphylococci* – interacts with -(1-3)- and -(1-4)-glucans – visual observation of colony color and morphology	+ manageable in high throughput and low cost
**Precipitation**
Precipitation Van Geel-Schutten et al. (1998)	*Screening of 182 LAB strains for EPS production:* – centrifugation of liquid culture precipitation with two volumes cold ethanol, stored overnight at 4C collection of precipitate by centrifugation dissolving in original volume, second precipitation drying at 55C and measuring the dry weight dissolving in original volume detection via phenol-sulfuric-acid-method and HPAEC-PAD	+ simple experimental setup and low cost + detailed monosaccharide analysis of selected strains difficult redissolving limited to EPS that can be precipitated time consuming
**Viscosity**
Culture viscosity Visual observation Garai-Ibabe et al. (2010)	*Screening of 147 LAB strains for* -*glucans*: EPS positive strains showed a ropy liquid culture and deposit formed a long string identification of the gene (*gtf*) encoding for -glucan-synthase -glucan agglutination test with *Streptococcus pneumoniae* type 37-specific antisera centrifugation of liquid culture precipitation with two volumes cold aceton and washing dissolving and detection via phenol-sulfuric acid method EPS characterization via NMR studies	+ preselection: simple experimental setup and low cost + specific identification of the -glucan-synthase gene + specific -glucan immunoprecipitation via antisera + NMR studies of selected EPS preselection via visual observation may result in many false negative (viscosity) molecular characterization is time consuming only EPS which precipitate were detected only determination of total carbohydrate content
Microhaematocrit capillaries Ricciardi et al. (1997)	– inversing tubes and measuring the time taken by the liquid to reach by gravity the opposite extremity of the tube precipitation with three volumes cold ethanol dissolving and detection via phenol-sulfuric acid method	+ simple experimental setup and low cost + only small volume needed manual handling only viscous EPS are detected
**Carbohydrate screening**
Uronic acid determination with m-hydroxydiphenyl Mojica et al. (2007)	*Screening for biofilm formation:* – dissolving of washed and dried biofilms intense vortexing for 2.5 min, 2 min resting adding reagent solution 1, vortex 45 s	+ manageable in high throughput + fast determination of UA + no interference with neutral sugars
	>– heating 100°C for 5 min adding reagent solution 2, vortex absorbance read at 520 nm after 4 min	– only detection of UA no discrimination of UA’s different color development for ManUA, GalUA, GlcUA
Modular exopolysaccharide screening platform Rühmann et al. (2015b)	* HT-Screening of 96-strains per day* – 96-well cultivation and cell removal visual observation of viscosity and precipitation assay 96-well gel-filtration and hydrolysis PMP-derivatization and UHPLC-UV-ESI-MS analysis	+ combination of different detection systems + detailed carbohydrate fingerprint + high throughput method only aldoses can be detected as ketoses cannot be derivatized with PMP

## Screening Approaches for Solid Media

### Detection of EPS Producing Phenotypes

Exopolysaccharides producers can be identified by their phenotypes on solid as well as liquid media. This technique is the most prevalent method to date and has been successfully used within the past several years to identify bacteria that are used for EPS production today. Generally, the terms “ropy,” “mucoid,” and “slimy” are used for this visual characterization. “Ropy” in liquid cultures is characterized via high resistance to flow through serological pipettes as well as via formation of viscous strands during “free fall” from the pipette tip (Vedamuthu and Neville, 1986). Furthermore, “ropy” colonies form long filaments when extended with an inoculation loop (Dierksen et al., 1997). The “mucoid” colonies have a glistening and slimy appearance on agar plates and do not form a filament during this process. One successful example for a screening via “mucoid” and “slimy” morphology was performed by Ortega-Morales et al. (2007) for a prescreening. Biomass of positive, “mucoid” and “slimy” strains was then scraped from the agar plate surface and diluted, before cells were removed and EPS was precipitated. The problem of this method is that it leads to false negative strains, which are excluded. After dissolving the precipitate the total carbohydrate content was determined via phenol-sulfuric-acid-method. The advantage of this screening method is that it can be easily performed without the need of any special equipment. The weakness of the method is that strain selection via colony morphology occurs by human interpretation and can hardly be standardized. Novel interesting polymers might not be detected due to a missing obvious slime formation. Ruas-Madiedo and De Los Reyes-Gavilán (2005) also pointed out that the nomenclature used to describe the different EPS producing phenotypes of lactic acid bacteria (LAB) can be confusing. The terms “ropy,” “mucoid” and “slimy” have been used indistinctly in literature, without any consequence and therefore lack of a clear definition.

A good way to evaluate mucoid and slimy colonies is the comparison of colony morphology between induced and non-induced EPS production. This can be efficiently used for strains showing extracellular sucrase activity, which is known to be inducible and strongly substrate dependent. Therefore, sucrose and raffinose as supplemented to the agar plates induce glucan- and fructansucrase activity of the EPS producers. Malik et al. (2009) screened 63 LAB-strains with this method and identified 29 isolates, from which 18 were randomly selected and proven via PCR to carry sucrase genes by use of degenerated primers. Tallgren et al. (1999) screened 600 strains for mucoid and slimy phenotypes and identified 170 interesting strains. Of these they only selected 10% (randomly chosen 17 strains) for further characterization of the monomeric composition, since no fast and reliable HT-screening methods for the determination of the monomeric composition of the produced polymers was available.

### Agar-Plates with Dyes

Some dyes are known to interact with polysaccharides with different specificities. This phenomenon can be used to identify different EPS-producers by a fast and easy agar plate based screening approach. Aniline Blue fluorochrome (Sinofluor) for example shows an intense fluorescence when bound to β-(1-3)-glucans. Additionally, the relative fluorescence with different types of other polysaccharides is well studied (Evans et al., 1984). Ma and Yin (2011) screened for EPS producing bacteria from different environments on LB-agar-plates supplemented with aniline blue. They identified 89 EPS producing strains and selected eight of them for further physiological, biochemical, and genetic analysis. The same technique, but with a different dye, was successfully utilized for the identification of EPS-defective mutants of *Rhizobium meliloti* (Leigh et al., 1985). Calcofluor White binds to succinoglycan as well as pure β-(1-3)- and β-(1-4)-glucans and exhibits a blue-green fluorescence when irradiated by long-wave UV light. Thereby, fluorescence negative colonies can easily be identified on agar-plates. This makes this method additionally suitable for a fast screening and characterization of a large number of mutagenized strains. Furthermore, there exist several dyes for various applications. Congo Red, for example, is known to interact with β-(1-3)- and β-(1-4)-glucans (Wood and Fulcher, 1978) and was successfully used to identify biofilm formation by different *Staphylococci* strains (Darwish and Asfour, 2013).

The use of dyes can be very useful when working with one or two defined polymers or if the screening targets a specific class of polysaccharide, e.g., β-(1-3)-glucan. However, screening for novel polymers containing different sugars, uronic acids as well as deoxy-and amino-sugars cannot be performed by use of these dyes, since interactions of the dyes to new EPS are unpredictable rendering this technique useless to identify novel EPS variants.

## Screening Approaches for Liquid Media

### Precipitation

When screening approaches are carried out in liquid media instead of solid media different techniques for EPS identification are required. Most EPS are highly soluble in aqueous solutions, whereas the solubility can be drastically decreased by using water miscible solvents by extracting water molecules from the hydration shell. Accordingly, for various EPS (xanthan gum, gellan gum, welan gum, diutan gum, succinoglycan, colanic acid) precipitation with alcohols or acetone is a common purification and isolation method (Phillips and Williams, 2000). In the same way it can also be applied for identification of EPS in screening approaches. The efficiency of precipitation of polymers depends on their chemical structure, molecular weight, and the final concentration of polymer and alcohol used for precipitation (Smidsroed and Haug, 1967; Swennen et al., 2005). Most importantly, it has to be taken into account that other biopolymers like DNA, RNA, proteins, and polyglutamat are also precipitating in the same manner (Schmid et al., 2013; Kreyenschulte et al., 2014). The appearance of the precipitate can help to distinguish the different polymers. Polysaccharides usually precipitate as fibers, when alcohols such as ethanol or 2-propanol are used as precipitant. However, this has to be considered with care as some EPS, like, e.g., hyaluronic acid precipitate more in the form of flakes.

Van Geel-Schutten et al. (1998) screened 182 LAB strains in de Man, Rogosa, and Sharpe (MRS) medium supplemented with different sugars. After 3 days of incubation at 37°C, the cells were removed from the cultures via centrifugation and EPS were precipitated with cold ethanol. This prescreening identified 60 strains that showed pellet formation. These were dried at 55°C, dissolved in water and the EPS-content was determined by measuring the total carbohydrate content with the phenol-sulfuric-acid-method (Dubois et al., 1956) and a detailed monosaccharide analysis for 17 selected strains was performed. However, one drawback of this method is that not all polysaccharides precipitate under the same conditions with alcohols or acetone (Sutherland, 1990; Azeredo and Oliveira, 1996). This has to be taken into account as limiting factor in the search for novel EPS structures, when only the precipitate is used for further analysis. On the other hand, the precipitation process is fast, easy to handle and at the same time purifies the EPS concerning remaining sugars and salts of the cultivation media (Kumar et al., 2007). This enables a better quantification of the total sugar content of the EPS and the polymer concentration can be increased by using less volume for dissolving the precipitate. This can lead to a higher sensitivity in the subsequent analysis. When the precipitate is dried after the supernatant is removed, the dry weight of the polymer can be determined to compare the productivity of different strains. However, in combination with HT-screening approaches, determination of the polymer dry weight is not applicable due to the use of multi-well format. Generally, an additional disadvantage of drying the precipitated polymer is an altered dissolving property of dried EPS, leading to inhomogeneous suspensions and therefore, to non-reliable results in subsequent analytics. Xu et al. (2010) screened 60 LAB strains in a similar way, but without drying the precipitate and without performing a detailed monosaccharide analysis. In this screening all LAB’s demonstrated their ability to produce EPS in different amounts. Only the strain showing the highest productivity was selected to be further optimized and characterized.

Taken together, precipitation is a fast and easy way to detect, isolate and purify polysaccharides. However, it is unsuitable for HT. It is difficult to handle precipitation in small volumes and even more though it is challenging to ensure the correct dissolving of the different precipitates (with or without drying) in 96-well format.

### Viscosity

Visually observable properties such as viscosity of the liquid culture have also been used for the evaluation of EPS production. A rapid screening for β-glucans based on this technique was performed by Garai-Ibabe et al. (2010). All strains that showed “ropy” liquid culture and had a long string formed by the cell deposit were selected. This criterion was used for a preselection to identify strains from which genes encoding β-glucansynthases could be amplified. However, not all viscous cultures show “ropy” behavior and sometimes there is just no correlation between phenotypic characterization on the one side and viscous behavior in liquid cultures on the other side Another reliable method is the determination of texture properties for example of LAB via a sensory and rheological screening (Folkenberg et al., 2006). These techniques for LAB and all other bacterial strains are very time consuming, though. Up to now there are no HT-screening applications in this field. Ricciardi et al. (1997) developed a rapid and convenient screening method for viscosity of LAB cultures based on the measurement of eﬄux time in microhaematocrit capillaries. For the simple experimental setup only small volumes of the samples are needed. However, the manual handling of this approach makes it more difficult to be manageable in HT.

### Specific Carbohydrate Screening

One possibility to search for specific carbohydrates is the targeted screening for uronic acids in biofilms (Mojica et al., 2007). This method is based on the method of Blumenkrantz and Asboe-Hansen (1973). The combination of hydrolyzing the sample and the specific color reaction caused by the uronic acids present in the polymer leads to a method capable for HT-applications. Mannuronic-, glucuronic-, and galacturonic-acids show different calibration curves and therefore, can be quantified reliably when only one known uronic acid is present in the biofilm. Discrimination of various uronic acids cannot be performed. However, this method presents a rapid screening for uronic acids in biopolymers without interference of neutral sugars.

To deal with all these issues, Rühmann et al. (2015b) recently developed a EPS screening platform, that combines visual observation of viscosity, and precipitation with a detailed monosaccharide analysis via UHPLC-UV-ESI-MS. All steps are handled in 96-well format, starting from the strain cultivation up to the carbohydrate fingerprint. Cells are removed via centrifugation and filtration steps and polymers are purified by removal of remaining low molecular compounds such as glucose and salts by gel-filtration. An aliquot of the gel-filtrate is then hydrolyzed, derivatized, and analyzed via an optimized HT-PMP method (HT-1-phenyl-3-methyl-5-pyrazolone; Rühmann et al., 2014). This method allows the simultaneous analysis of hexoses, pentoses, deoxy, and amino-sugars, uronic acids as well as different sugar modifications in one single run. Double detection via UV- and ESI-MS-detector makes that quantification very reliable. Additionally, the method is also very fast. The carbohydrate fingerprint of 96 different strains can be analyzed within only 1 day. Other detection modules like precipitation and visual observation of viscosity are handled in parallel and therefore, there is not only one screening method causing a preselection. This allows to trap the full potential of various EPS producers. Additionally, the carbohydrate fingerprint enables a targeted screening. The throughput can be even increased by the factor of eight when coupling the detailed carbohydrate fingerprint analysis with a modular automated prescreening system (Rühmann et al., 2015a).

## 96-Well Colorimetric Carbohydrate Assays

Usually, methods for the determination of the total carbohydrate content are combined with other screening methods as shown in **Table 1**. Mostly they are placed at the end of the screening approach and for that reason will not be applied to all strains. However, to grab the full potential of all the different polysaccharides it would be more suitable to place the determination of total carbohydrate content as first detection system for EPS in order not to produce false negatives. **Table 2** gives an overview of some 96-well micro-titer-plate (MTP) based methods for the rapid analysis of carbohydrate content in parallel. The phenol-sulfuric-acid-method still represents the common procedure for the fast determination of total carbohydrate content of bacterial and plant polysaccharides (Ortega-Morales et al., 2007; Xu et al., 2010). This method was first described by Dubois et al. (1956) and later adapted to a 96-well application (Masuko et al., 2005). Masuko et al. (2005) optimized it toward a really rapid method (<15 min) with only one heating step and without any mixing step. They reached a linear calibration range from 4.5 to 676 mg/L with a coefficient of determination (*R*^2^) of 0.988. The major disadvantages of this method are the need of a carcinogenic phenol reagent and the incubation in a water bath, what necessitates manual drying of the plate. In addition the method does not provide real quantitative values as the molar extinction coefficient of different sugars varies causing unequal responses of different heteropolysaccharides.

**Table 2:** Overview and description of different micro-titer based colorimetric assays including benefits and disadvantages.

Colorimetric assay/ Reference	Description	Calibration range/*R*^2^	Pros and Cons
**Phenol-sulfuric-acid** Masuko et al. (2005)	50 μL sample + 150 μL concentrated sulfuric acid + 30 μL 5% phenol (w/v) Incubation: 5 min at 90°C in a water bath, cooling down Measurement: at 490 nm	4.5–676 mg/L 0.988 *R*^2^	+ Very fast (<15 min) + No mixing step + One heating step Carcinogenic Manual drying of the plates after incubation in the water bath
**Sulfuric-acid w/o phenol** Albalasmeh et al. (2013)	1 mL sample + 3 mL of concentrated sulfuric acid rapidly mixed in a test tube Shaking: vortex for 30 s Measurement: at 315 nm	10–70 mg/L 0.992 *R*^2^	+ Non-carcinogenic phenol + Reduced measurement time and error + No extra heating step Not tested for high throughput in micro titer plates Small linear calibration range Interference by protein and/or flavonoid impurities
**Anthrone-sulfuric-acid** Laurentin and Edwards (2003)	40 μL sample, cover plate with cling film, vortex gently Incubation: 15 min at 4°C (preparing reagent) 100 μL reagent (2 g/L anthrone in concentrated sulfuric acid), seal with plate sealer, vortex gently Incubation: 3 min at 92°C; 5 min at RT; 15 min at 45°C Measurement: at 630 nm	50–400 mg/L 0.982 *R*^2^	+ Non-carcinogenic + Selective to hexoses Requirements needed (e.g., cling film, acetat film) Two heating steps 11 main steps for preparing the assay Time consuming preparation (∼2.5 h)
**Carbazole** Cesaretti et al. (2003)	50 μL sample, 200 μL (25 mM sodium tetraborate in sulfuric acid) Incubation: 10 min at 100°C in an oven, cooling down 15 min Carefully adding 50 μL (0,125% carbazole in ethanol) Incubation: 10 min at 100°C in an oven; cooling down Measurement: at 550 nm	20–2,000 mg/L 0.975 *R*^2^	+ Detection of glycosaminoglycans (e.g., heparin, chondroitin and hyaluronic acid) Two heating steps Long preparation time (∼1 h) Low interferences with hexoses
***m*-Hydroxydiphenyl** Cesaretti et al. (2003)	40 μL sample + 200 μL (120 mM sodium tetraborate in sulfuric acid) Incubation: 1 h at 80°C Measurement: at 540 nm (background) 40 μL reagent (100 μL (100 mg/mL m-hydroxydiphenyl in dimethyl sulfoxide) mixed with 4.9 mL 80% (v/v) sulfuric acid just before use) Incubation: 15 min at room temperature Measurement: at 540 nm	12.5–200 mg/L	+ Detection of glycosaminoglycans + Background subtraction + No interference of 20-fold excess of neutral sugar to uronic acids + Lower hydrolysis temperature Long preparation time (∼1.5 h) Small linear calibration range
**Phenol-sulfuric-acid coupled with glucose-assay** Rühmann et al. (2015a)	20 μL gel-filtrate + 180 μL phenol-sulfuric-acid (30 μL 5% (w/v) phenol in ddH_2_O + 150 μL concentrated sulfuric-acid, mixed before on ice) Shaking: 5 min at 900 rpm Incubation: 35 min at 80°C in an oven; cooling down Measurement: at 480 nm	50–5,000 mg/L 0,999 *R*^2^	+ Background subtraction of remaining glucose after cultivation via glucose assay One mixing step Medium preparation time (∼1 h)

Albalasmeh et al. (2013) developed a non-carcinogenic assay by removing the phenolic component. They also reduced the reaction time to a minimum and eliminated the heating step. By this setup they reduced the error in measurement and developed a more reliable method. This simple approach would be easily transferred to an automated liquid handling station to perform an HT-screening. However, a MTP version of the method was not reported up to now and has to be validated first. Additionally, the lack of phenol eliminates the ability to produce a color reaction and therefore, the UV absorbance at 315 nm strongly interferes with protein and/or flavonoid impurities.

Another non-carcinogenic assay was developed by Laurentin and Edwards (2003). This group used anthrone as reaction chemical, which mainly interacts with hexoses, and shows only slight reactivity with pentoses, or uronic acids. However, the limit of quantification (LOQ) is 10 times lower than for the phenol-sulfuric-acid-method. Furthermore, special equipment as well as many additional and time consuming handling steps are necessary to perform this assay.

Cesaretti et al. (2003) reported an MTP based assay with carbazole to detect uronic acids in glycosaminoglycans. The uronic acids were stabilized with tetraborate and hydrolyzed via sulfuric-acid in the first incubation step. The color reaction caused by carbazole takes place during a second incubation step. The method showed the lowest value for the coefficient of determination (*R*^2^) with 0.975 and at the same time the widest linear calibration range (20–2,000 mg/L) of all methods as summarized in **Table 2**. Another assay for the determination of glycosaminoglycans was reported by Van Den Hoogen et al. (1998). The hydrolysis in the first step of this method is similar to the first step of the carbazole assay. Afterward, the brownish background color reaction caused by neutral sugars was measured at 540 nm. In the second incubation step the color reaction with *m*-hydroxydiphenyl and uronic acids takes place at room temperature and once again the absorbance was measured at 540 nm. The background subtraction enables a robust method where an excess of neutral sugars (up to 20-fold) does not interfere with the determination of the uronic acids. Both described assays are capable for targeted screening approaches for uronic acid containing polymers.

However, when screening for various kinds of polymers only total carbohydrate content methods are appropriate. They are able to detect all kinds of sugars and determine their total content, but are not able to distinguish between different monomeric, oligomeric, or polymeric carbohydrates within the samples. Taking this into account, the choice of a suitable cultivation medium is essential to obtain reliable results. Complex media containing oligomeric or polymeric carbohydrate compounds (like, e.g., yeast extract) might lead to falsely increased values. Therefore, these complex media compounds should be avoided. Remaining carbohydrates from the cultivation process can also negatively interfere with the measurement of the EPS content, since often high amounts of sugars are used as C-source for cultivation of EPS producing strains. Therefore, a precipitation step is usually necessary to separate the monomeric and oligomeric material from the polymeric precipitate. After precipitation the dissolved polymer can be applied to further measurements. However, both steps, precipitation and dissolving, are hardly suitable for HT-screening approaches. A different possibility would be to eliminate oligomeric substances in the media, to determine the remaining monomeric carbohydrates and to subtract this value from the total carbohydrate content. Ruijssenaars et al. (2000) investigated the biodegradability of extracellular polysaccharides and therefore, calculated the EPS content. This was done by measuring the total carbohydrate content via phenol-sulfuric-acid-method and subtraction of the reducing sugar concentration determined with dinitrosalicylic-acid (DNS) method (Miller, 1959). Another group adapted the DNS method toward a 96-well format (Negrulescu et al., 2012). Generally, the determination of reducing sugars is of special interest for the saccharification analysis of biomass and massive research is performed in this field. Whitehead et al. (2012) reported a completely automated analysis of reducing sugars via 3-methyl-2-benzothiazolinone hydrazone (MBTH). Other methods use enzymatic glucose oxidation, coupled with a color reaction to determine the remaining glucose after cultivation (Rühmann et al., 2015a). In combination with the total carbohydrate content this method enables the selective detection of EPS without precipitation and dissolving. This method is designed for automated handling as well as for manual execution in 96-well format.

## Conclusion

Different techniques for EPS screening approaches are available. Identification of EPS via colony morphology, dye based plate-assays, precipitation, and visual evaluation of viscosity are techniques, which do not require special equipment. They all have their own benefits and limitations, which have to be taken into account. During the past few years colorimetric assays have been more and more transferred to 96-well format to determine different sugars in various applications and to increase the throughput. Further development in the carbohydrate analysis via UHPLC and MS technology enables a fast and reliable determination of the carbohydrate fingerprint, which gives a more detailed overview of the monomeric composition. Additionally, automatization platforms are available and even further increase the throughput for screening of various strains. However, until now there are no HT-screening approaches available for the determination of the molecular weight distribution and/or rheological properties of microbial polymers. Development of such techniques might be hard to realize, but would drastically enhance our knowledge on microbial EPS. Other techniques, such as NMR, already give further information about the sequence of the repeating unit including linkages in-between these heteropolysaccharides. As soon as they are available in HT this would revolutionize the targeted screening of EPS.

## Conflict of Interest Statement

The authors declare that the research was conducted in the absence of any commercial or financial relationships that could be construed as a potential conflict of interest.

## References

[B1] Albalasmeh A. A. Berhe A. A. Ghezzehei T. A. (2013). A new method for rapid determination of carbohydrate and total carbon concentrations using UV spectrophotometry. *Carbohydr. Polym.* 97 253–261. 10.1016/j.carbpol.2013.04.072 23911443

[B2] Azeredo J. Oliveira R. (1996). A new method for precipitating bacterial exopolysaccharides. *Biotechnol. Tech.* 10 341–344. 10.1007/BF00173251

[B3] Blumenkrantz N. Asboe-Hansen G. (1973). New method for quantitative determination of uronic acids. *Anal. Biochem.* 54 484–489. 10.1016/0003-2697(73)90377-1 4269305

[B4] Cesaretti M. Luppi E. Maccari F. Volpi N. (2003). A 96-well assay for uronic acid carbazole reaction. *Carbohydr. Polym.* 54 59–61. 10.1016/S0144-8617(03)00144-9

[B5] Colegrove G. T. (1983). Agricultural applications of microbial polysaccharides. *Industrial Eng. Chem. Prod. Res. Dev.* 22 456–460. 10.1021/i300011a014

[B6] Costerton J. W. Stewart P. S. Greenberg E. P. (1999). Bacterial biofilms: a common cause of persistent infections. *Science* 284 1318–1322. 10.1126/science.284.5418.1318 10334980

[B7] Darwish S. F. Asfour H. A. E. (2013). Investigation of biofilm forming ability in Staphylococci causing bovine mastitis using phenotypic and genotypic assays. *Sci. World J.* 2013 378492 10.1155/2013/378492 PMC3835882 24298212

[B8] Dierksen K. P. Sandine W. E. Trempy J. E. (1997). Expression of ropy and mucoid phenotypes in *Lactococcus lactis*. *J. Dairy Sci.* 80 1528–1536. 10.3168/jds.S0022-0302(97)76082-X 9276790

[B9] Dubois M. Gilles K. A. Hamilton J. K. Rebers P. A. Smith F. (1956). Colorimetric method for determination of sugars and related substances. *Anal. Chem.* 28 350–356. 10.1021/ac60111a017

[B10] Evans N. A. Hoyne P. A. Stone B. A. (1984). Characteristics and specificity of the interaction of a fluorochrome from aniline blue (Sirofluor) with polysaccharides. *Carbohydr. Polym.* 4 215–230. 10.1016/0144-8617(84)90012-2

[B11] Folkenberg D. M. Dejmek P. Skriver A. Skov Guldager H. Ipsen R. (2006). Sensory and rheological screening of exopolysaccharide producing strains of bacterial yogurt cultures. *Int. Dairy J.* 16 111–118. 10.1016/j.idairyj.2004.10.013

[B12] Garai-Ibabe G. Areizaga J. Aznar R. Elizaquivel P. Prieto A. Irastorza A. (2010). Screening and selection of 2-Branched (13)-β-d-glucan producing lactic acid bacteria and exopolysaccharide characterization. *J. Agric. Food Chem.* 58 6149–6156. 10.1021/jf904529q 20423154

[B13] Kreyenschulte D. Krull R. Margaritis A. (2014). Recent advances in microbial biopolymer production and purification. *Crit. Rev. Biotechnol.* 34 1–15. 10.3109/07388551.2012.743501 23190337

[B14] Kumar A. S. Mody K. Jha B. (2007). Bacterial exopolysaccharides – a perception. *J. Basic Microbiol.* 47 103–117. 10.1002/jobm.200610203 17440912

[B15] Laurentin A. Edwards C. A. (2003). A microtiter modification of the anthrone-sulfuric acid colorimetric assay for glucose-based carbohydrates. *Anal. Biochem.* 315 143–145. 10.1016/S0003-2697(02)007042 12672425

[B16] Leigh J. A. Signer E. R. Walker G. C. (1985). Exopolysaccharide-deficient mutants of *Rhizobium meliloti* that form ineffective nodules. *Proc. Natl. Acad. Sci. U.S.A.* 82 6231–6235. 10.1073/pnas.82.18.6231 3862129 PMC391026

[B17] Ma J.-J. Yin R.-C. (2011). Primary study on extracellular polysaccharide producing bacteria in different environments. *Anhui Daxue Xuebao Ziran Kexueban* 35 94–100.

[B18] Malik A. Radji M. Kralj S. Dijkhuizen L. (2009). Screening of lactic acid bacteria from Indonesia reveals glucansucrase and fructansucrase genes in two different *Weissella confusa* strains from soya. *FEMS Microbiol. Lett.* 300 131–138. 10.1111/j.1574-6968.2009.01772.x 19758326

[B19] Masuko T. Minami A. Iwasaki N. Majima T. Nishimura S.-I. Lee Y. C. (2005). Carbohydrate analysis by a phenol-sulfuric acid method in microplate format. *Anal. Biochem.* 339 69–72. 10.1016/j.ab.2004.12.001 15766712

[B20] Miller G. L. (1959). Use of dinitrosalicylic acid reagent for determination of reducing sugar. *Anal. Chem.* 31 426–428. 10.1021/ac60147a030

[B21] Mojica K. Elsey D. Cooney M. J. (2007). Quantitative analysis of biofilm EPS uronic acid content. *J. Microbiol. Methods* 71 61–65. 10.1016/j.mimet.2007.07.010 17822791

[B22] Negrulescu A. Patrulea V. Mincea M. M. Ionascu C. Vlad-Oros B. A. Ostafe V. (2012). Adapting the reducing sugars method with dinitrosalicylic acid to microtiter plates and microwave heating. *J. Braz. Chem. Soc.* 23 2176–2182. 10.1590/S0103-50532013005000003

[B23] Ortega-Morales B. O. Santiago-García J. L. Chan-Bacab M. J. Moppert X. Miranda-Tello E. Fardeau M. L. (2007). Characterization of extracellular polymers synthesized by tropical intertidal biofilm bacteria. *J. Appl. Microbiol.* 102 254–264. 10.1111/j.1365-2672.2006.03085.x 17184342

[B24] Phillips G. O. Williams P. A. (2000). “Preface,” in *Handbook of Hydrocolloids* eds Phillips G. O. Williams P. A. (Abington, MA: Wooddead Publishing Limited).

[B25] Prajapati V. D. Jani G. K. Zala B. S. Khutliwala T. A. (2013). An insight into the emerging exopolysaccharide gellan gum as a novel polymer. *Carbohydrate Polymers* 93 670–678. 10.1016/j.carbpol.2013.01.030 23499110

[B26] Rau U. Brandt C. (1994). Oxygen controlled batch cultivations of *Schizophyllum* commune for enhanced production of branched β-13-glucans. *Bioprocess Eng.* 11 161–165. 10.1007/BF00518738

[B27] Ricciardi A. Parente E. Clementi F. (1997). A simple method for the screening of lactic acid bacteria for the production of exopolysaccharides in liquid media. *Biotechnol. Techn.* 11 271–275. 10.1023/A:1018450824524

[B28] Ruas-Madiedo P. De Los Reyes-Gavilán C. G. (2005). Invited review: methods for the screening, isolation, and characterization of exopolysaccharides produced by lactic acid bacteria. *J. Dairy Sci.* 88 843–856. 10.3168/jds.S0022-0302(05)72750-8 15738217

[B29] Rühmann B. Schmid J. Sieber V. (2014). Fast carbohydrate analysis via liquid chromatography coupled with ultra violet and electrospray ionization ion trap detection in 96-well format. *J. Chromatogr. A* 1350 44–50. 10.1016/j.chroma.2014.05.014 24861788

[B30] Rühmann B. Schmid J. Sieber V. (2015a). Automated modular high throughput exopolysaccharide screening platform coupled with highly sensitive carbohydrate fingerprint analysis. *J. Visualized Exp.* 10.3791/53249 PMC4941912 27167303

[B31] Rühmann B. Schmid J. Sieber V. (2015b). High throughput exopolysaccharide screening platform: From strain cultivation to monosaccharide composition and carbohydrate fingerprinting in one day. *Carbohydrate Polymers* 122 212–220. 10.1016/j.carbpol.2014.12.021 25817661

[B32] Ruijssenaars H. J. Stingele F. Hartmans S. (2000). Biodegradability of food-associated extracellular polysaccharides. *Curr. Microbiol.* 40 194–199. 10.1007/s002849910039 10679053

[B33] Schmid J. Mueller-Hagen D. Sieber V. Meyer V. (2013) “Nucleic and protein extraction methods for fungal exopolysaccharide producers,” in *Laboratory Protocols in Fungal Biology* eds Gupta V. K. Tuohy, M. G. Ayyachamy, M. Turner, K. M. O’donovan A. (New York: Springer), 427–434 10.1007/978-1-4614-2356-0_39

[B34] Schmidt W. Brouwers H. J. H. Kuehne H.-C. Meng B. (2013). The working mechanism of starch and diutan gum in cementitious and limestone dispersions in presence of polycarboxylate ether superplasticizers. *Appl. Rheol.* 23 52903–52914. 10.3933/ApplRheol-23-52903

[B35] Smidsroed O. Haug A. (1967). Precipitation of acidic polysaccharides by salts in ethanol-water mixtures. *J. Polym. Sci. Polym. Lett. Ed.* 16 1587–1598. 10.1002/polc.5070160335

[B36] Srinivasan R. (2013). “Natural polysaccharides as treatment agents for wastewater,” in *RSC Green Chemistry Ser.* eds Mishra A. Clark J. H. (Cambridge: Royal Society of Chemistry), 51–81.

[B37] Sutherland I. W. (1990). *Biotechnology of Microbial Exopolysaccharides*. Cambridge: Cambridge University Press 10.1017/CBO9780511525384

[B38] Swennen K. Courtin C. M. Van Der Bruggen B. Vandecasteele C. Delcour J. A. (2005). Ultrafiltration and ethanol precipitation for isolation of arabinoxylooligosaccharides with different structures. *Carbohydrate Polymers* 62 283–292. 10.1016/j.carbpol.2005.08.001

[B39] Tallgren A. H. Airaksinen U. Von Weissenberg R. Ojamo H. Kuusisto J. Leisola M. (1999). Exopolysaccharide-producing bacteria from sugar beets. *Appl. Environ. Microbiol.* 65 862–864. 9925632 10.1128/aem.65.2.862-864.1999 PMC91111

[B40] Thibodeau A. (2005). Protecting the skin from environmental stresses with an exopolysaccharide formulation. *Cosmet. Toiletries* 120 81–82, 84–90.

[B41] Toukach P. Joshi H. J. Ranzinger R. Knirel Y. Von Der Lieth C.-W. (2007). Sharing of worldwide distributed carbohydrate-related digital resources: online connection of the Bacterial Carbohydrate Structure DataBase and GLYCOSCIENCES.de. *Nucleic Acids Res.* 35 D280–D286. 10.1093/nar/gkl883 17202164 PMC1899093

[B42] Van Den Hoogen B. M. Van Weeren P. R. Lopes-Cardozo M. Van Golde L. M. G. Barneveld A. Van De Lest C. H. A. (1998). A microtiter plate assay for the determination of uronic acids. *Anal. Biochem.* 257 107–111. 10.1006/abio.1997.2538 9514779

[B43] Van Geel-Schutten G. H. Flesch F. Ten Brink B. Smith M. R. Dijkhuizen L. (1998). Screening and characterization of *Lactobacillus* strains producing large amounts of exopolysaccharides. *Appl. Microbiol. Biotechnol.* 50 697–703. 10.1007/s002530051353

[B44] Vedamuthu E. R. Neville J. M. (1986). Involvement of a plasmid in production of ropiness (mucoidness) in milk cultures by *Streptococcus cremoris* MS. *Appl. Environ. Microbiol.* 51 677–682. 16347031 10.1128/aem.51.4.677-682.1986 PMC238946

[B45] Whitehead C. Gomez L. D. Mcqueen-Mason S. J. (2012). The analysis of saccharification in biomass using an automated high-throughput method. *Methods Enzymol.* 510 37–50. 10.1016/B978-0-12-415931-0.00003 22608720

[B46] Wood P. J. Fulcher R. G. (1978). Interaction of some dyes with cereal β-glucans. *Cereal Chem.* 55 952–966.

[B47] Xu R. Ma S. Wang Y. Liu L. Li P. (2010). Screening, identification and statistic optimization of a novel exopolysaccharide producing *Lactobacillus paracasei* HCT. *Afr. J. Microbiol. Res.* 4 783–795.

